# Assessment of Dentoalveolar Changes Following Leveling and Alignment of Severely Crowded Upper Anterior Teeth Using Self-Ligating Brackets Alone or With Flapless Piezocision Compared to Traditional Brackets: A Randomized Controlled Clinical Trial

**DOI:** 10.7759/cureus.35733

**Published:** 2023-03-03

**Authors:** Heba M Al-Ibrahim, Mohammad Y Hajeer, Ahmad S Burhan, Yaser Haj Hamed, Issam Alkhouri, Eiad Zinah

**Affiliations:** 1 Department of Orthodontics, University of Damascus Faculty of Dentistry, Damascus, SYR; 2 Department of Orthodontics, Appolonia Pediatric Dentistry Center, Dubai, ARE; 3 Department of Oral and Maxillofacial Surgery, University of Damascus Faculty of Dentistry, Damascus, SYR; 4 Department of Dental Public Health, University College London, London, GBR

**Keywords:** canine rotation, intercanine width, traditional brackets, acceleration of tooth movement, piezocision, leveling, flapless corticotomy, self-ligating brackets, dentoalveolar changes

## Abstract

Introduction

Dental crowding is one of the most common types of malocclusions. It can be treated with or without extraction, depending on the severity of the crowding. Extraction-based orthodontic treatments are the preferred treatment option in cases of severe crowding, but they take longer than non-extraction cases.

Objective

This study aimed to evaluate the dentoalveolar changes following the orthodontic treatment of severely crowded maxillary anterior teeth in adults using self-ligating brackets alone or combined with flapless piezocision.

Materials and methods

The participants in this study were 63 patients (46 females and 17 males; mean age SD: 19.71 ± 2.74 years) who attended the Department of Orthodontics at the University of Damascus from January 2020 to December 2021. The participants were divided into three groups at random: Group (1): traditional brackets group, Group (2): self-ligating brackets group; and Group (3): self-ligating brackets with flapless piezocision group. Little's Irregularity Index (LII) was measured at five assessment times: before the onset of orthodontic treatment (T0), after one month (T1), after two months (T2), after three months (T3), and at the end of the leveling and alignment phase (T4). The intercanine width (lingual), the intercanine width (cusp), and the canine rotation angle were measured at two assessment times: before the onset of orthodontic treatment (T0) and at the end of the leveling and alignment phase (T4).

Results

The three studied groups had statistically significant differences in terms of LII during the first three months, and the most significant improvement of LII was in the self-ligating brackets with the piezocision group (P < 0.001). In addition, the intercanine width (cusp) at the end of the leveling and aligning phase revealed greater mean values in both self-ligating brackets groups compared to the traditional brackets group, and the differences were statistically significant (P < 0.001). Otherwise, no statistically significant differences were found at the end of the leveling and aligning phase in the intercanine width (lingual) or the canine rotation angle between the three studied groups (P > 0.05).

Conclusion

Using self-ligating brackets with flapless piezocision revealed more significant results concerning LII as compared to other groups. Thus, combining these two acceleration methods could get more effective results in aligning severely crowded teeth. Self-ligating brackets, whether used alone or with flapless piezocision, resulted in greater intercanine width at the cusp level. The type of brackets (traditional or self-ligating) did not affect the canine rotation angle.

## Introduction

Dental crowding is one of the most common types of malocclusion [1,2]. Depending on the severity of crowding, it can be treated with or without extraction [1,2]. Extraction-based orthodontic treatment is the preferred treatment option in cases of severe crowding, but they take longer than non-extraction cases [1,2].

Many patients avoid orthodontic treatment due to the length of the treatment period [3]. The prolonged period of orthodontic treatment increases the negative effects of fixed orthodontic appliances such as white spots, cavities, root resorption, gingival recession, and pain and discomfort [4-6]. Therefore, reducing the duration of orthodontic treatment is important for orthodontists and patients, especially adults, who want to finish their treatment in the shortest possible time [7,8].

Tooth movement depends on the application of orthodontic forces [3]. These forces induce many changes in the periodontal ligaments, resulting in bone and periodontal tissue remodeling [9]. Many factors influence tooth movement, including individual differences, age, type of malocclusion, treatment techniques used, and patient cooperation [7].

Many acceleration methods have been used to reduce the period of orthodontic treatment such as mechanical, surgical, and physical methods [10-12]. It has been believed that using self-ligating as a mechanical acceleration method reduces friction forces [13]. The orthodontic movements are affected by friction forces between the orthodontic wire and the bracket slot [1,14,15]. The friction force values differ according to the physical characteristics and dimensions of the brackets and wires used and the binding type [13,14,16,17]. Concerning the surgical methods, the orthodontic movement acceleration results mainly from the induction of the regional acceleratory phenomenon (RAP) [18-21].

Regarding the participation of self-ligating brackets with surgical acceleration procedures, the studies carried out so far are limited to only two [22,23]. The first one is Charavet et al.'s study, which included adult patients with mild to moderate crowding on the two jaws and focused on studying the duration of orthodontic treatment and the status of the periodontal tissues without studying the accompanying dentoalveolar changes [22]. The second study was conducted by Mittal et al., who limited their trial to investigate the acceleration of orthodontic movement following the application of micro-osteoperforations with self-ligating brackets in space closure without studying any other variables [23].

Therefore, no published paper has yet studied the dentoalveolar changes following the treatment of severely crowded maxillary anterior teeth using self-ligating brackets with flapless piezocision. Consequently, this randomized controlled trial was conducted to fill this medical literature gap. The null hypothesis assumes no differences in the dentoalveolar changes associated with self-ligating brackets, whether used alone or combined with flapless piezocision, compared to the traditional brackets in adults.

## Materials and methods

Study design and registration 

Between January 2020 and December 2021, the principal researcher examined patients enrolled in the Department of Orthodontics at Damascus University's Dental School. This study followed the Consolidated Standards of Reporting Trials (CONSORT) guidelines for writing this report. Local Research Ethics Committee approval was obtained from the University of Damascus (UDDS-478-16012020/SRC-5973). The Postgraduate Research Budget of Damascus University was responsible for funding the research project (Ref no: 46595420JDF).

Sample size calculation

Minitab® Version 18 (Minitab Inc., State College, Pennsylvania, USA) was the software used in sample size calculation. It was assumed that 1 mm with a standard deviation of 0.99 mm was the significant difference in the Little’s Irregularity Index (LII) according to the Jahanbin et al. study [17]. Utilizing the analysis of variance (ANOVA) test with a 5% significance level and 80% as the power of the study, 20 patients in each group were required. One participant was added to each group to prevent any possible attrition, resulting in 63 as the total study sample. 

Patients' recruitment and eligibility criteria

**Figure 1:**
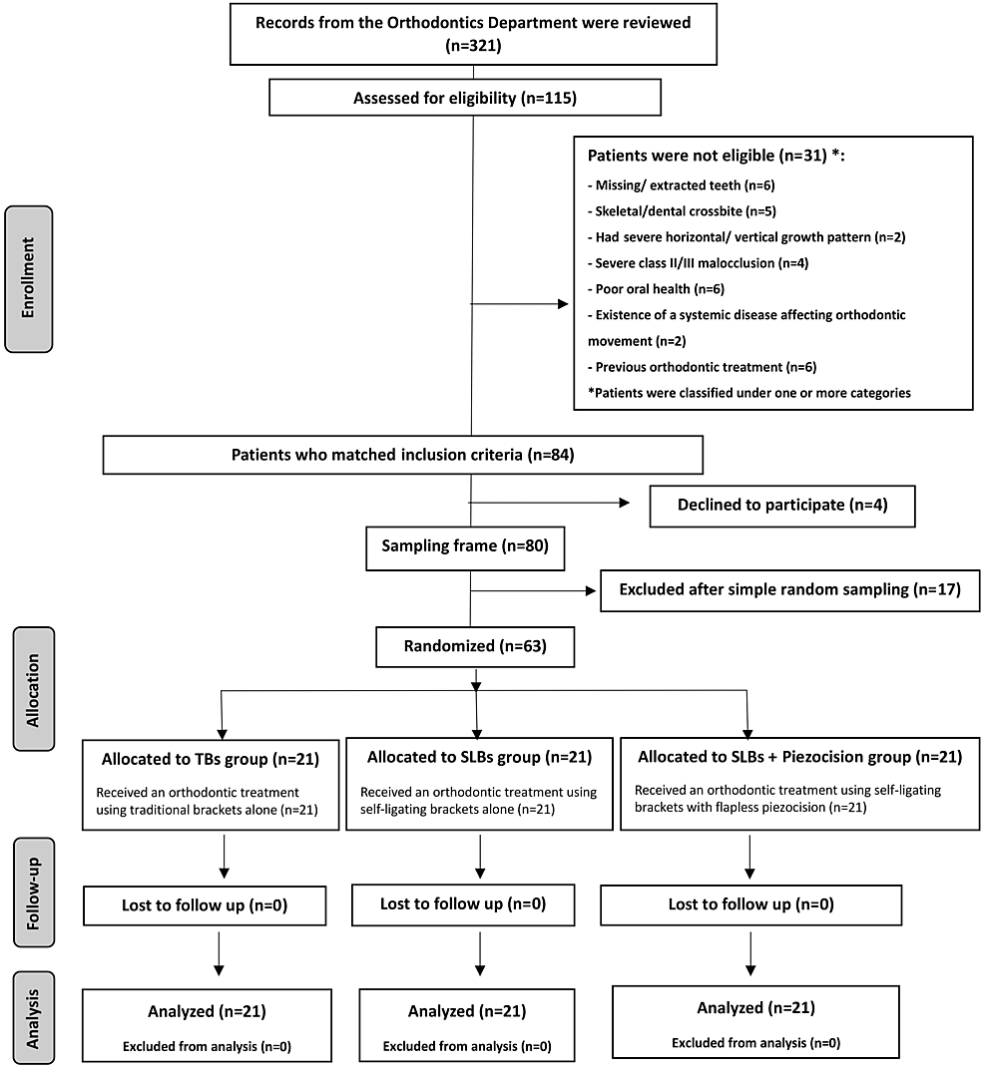
The Consolidated Standards of Reporting Trials (CONSORT) flow diagram of patients' recruitment, follow-up, and entry into data analysis

A clinical assessment of 115 patients at the University of Damascus, Department of Orthodontics, revealed that 84 patients met the selection criteria. After providing enough information to all patients regarding the orthodontic and surgical procedures planned for this study, 63 of the 80 individuals who agreed to participate were randomly chosen (Figure 1). All chosen patients received informational sheets before obtaining informed consent forms. The inclusion criteria were: (1) The patient is between the ages of 17 and 28, (2) Maxillary severe dental crowding greater than 6 mm, (3) Class I malocclusion, (4) LII exceeds 7 millimeters, and (5) No tooth loss. The exclusion criteria were: (1) The existence of a cleft lip and palate or any congenital disorders, (2) Skeletal or dental crossbite on the upper jaw, (3) Any systemic disorder affecting teeth movements, and (4) Previous orthodontic treatment. The CONSORT flow diagram of patients' recruitment, follow-up, and entry into data analysis is given in Figure 1.

Randomization, allocation concealment, and blinding 

Software called SPSS® Version 20 (SPSS for Windows, version 20, IBM Corp, Armonk, NY) was used to divide patients equally between the study groups. Before extracting the first premolars, a series of random numbers were used to apply the allocation concealment, and the allocation sequence was concealed using sealed envelopes. An academic person not involved in this trial carried out the random allocation sequence. The 63 patients were randomized into three groups: In Group (1), only traditional brackets were used for orthodontic treatment; in Group (2), only self-ligating brackets were used; and in Group (3), patients underwent piezocision-assisted orthodontic treatment using self-ligating brackets. It was not feasible to blind either the principal researcher (H.M.I.) or the participants, and the blinding was limited to the outcomes' assessor.

Orthodontic procedures

The orthodontic appliances consisted of traditional brackets (Master Series®, American Orthodontics, Sheboygan, Wisconsin) or active self-ligating brackets (Empower2®, American Orthodontics) with slot dimensions of 0.022 x 0.028 inch. The system of brackets used for each patient was selected according to his/her group. The orthodontic appliance was applied after a week of premolar extraction for all patients to prevent affecting the piezocision's results in the piezocision-assisted orthodontic treatment group. The following archwires sequences were conducted to perform leveling and alignment of the severely crowded teeth: 0.012-inch nickel-titanium (NiTi), 0.014-inch NiTi, 0.016-inch NiTi, 0.016*0.022-inch NiTi, 0.017*0.025-inch NiTi, 0.019*0.025-inch NiTi, 0.017*0.025-inch stainless steel (SS). The leveling and alignment phase was considered accomplished when 0.017*0.025-inch stainless steel (SS) wire could be inserted without applying an exaggerated force.

Surgical intervention

The surgical intervention was carried out at the Oral and Maxillofacial Surgery department, Damascus University, by the principal researcher (H.M.I), under the control of one of the supervisors (I.K). Before the surgical procedure, the participant was instructed to rinse for one minute with 0.12% chlorhexidine mouthwash (Oral-B, Procter & Gamble Company, Cincinnati, Ohio). After that, regional anesthesia in the incisive and infraorbital foramen was applied using 2% lidocaine with adrenaline 1:80,000 (New Stetic, Newcaina, Colombia). The surgical intervention initially included making vertical incisions in the soft tissue from both palatal and buccal sides using a no. 15 blade [2]. These incisions were placed 3 mm apical to the interdental papilla to prevent gingival recessions [2]. After that, cortical alveolar incisions ranging from 5 mm to 8 mm in length and 3 mm in depth were made using a piezosurgery knife with a BS1 tip (Implant Center™ 2, Satelec, France) [2]. One incision was conducted between every two roots of the six upper anterior teeth while three incisions were performed in each extracted first premolar region. 

The patient was instructed to follow a post-corticotomy regimen, including applying ice packs over the cheek area during the first 12 hours, consuming soft food for the first three days, using mouthwash for one week, and fully refraining from smoking [20]. Nonsteroidal anti-inflammatory medications were prohibited to avoid obstructing the acceleratory phenomenon [2]. Only 500 mg of paracetamol was permitted if necessary to avoid obstructing the acceleratory phenomenon [2].

Outcome measures

The outcome measures included LII, the intercanine width (lingual), the intercanine width (cusp), and the canine rotation angle. LII was measured at five assessment times: before the onset of orthodontic treatment (T0), after one month (T1), after two months (T2), after three months (T3), and at the end of the leveling and alignment phase (T4). The intercanine width (lingual), the intercanine width (cusp), and the canine rotation angle were measured at two assessment times: before the onset of orthodontic treatment (T0) and at the end of the leveling and alignment phase (T4).

**Figure 2:**
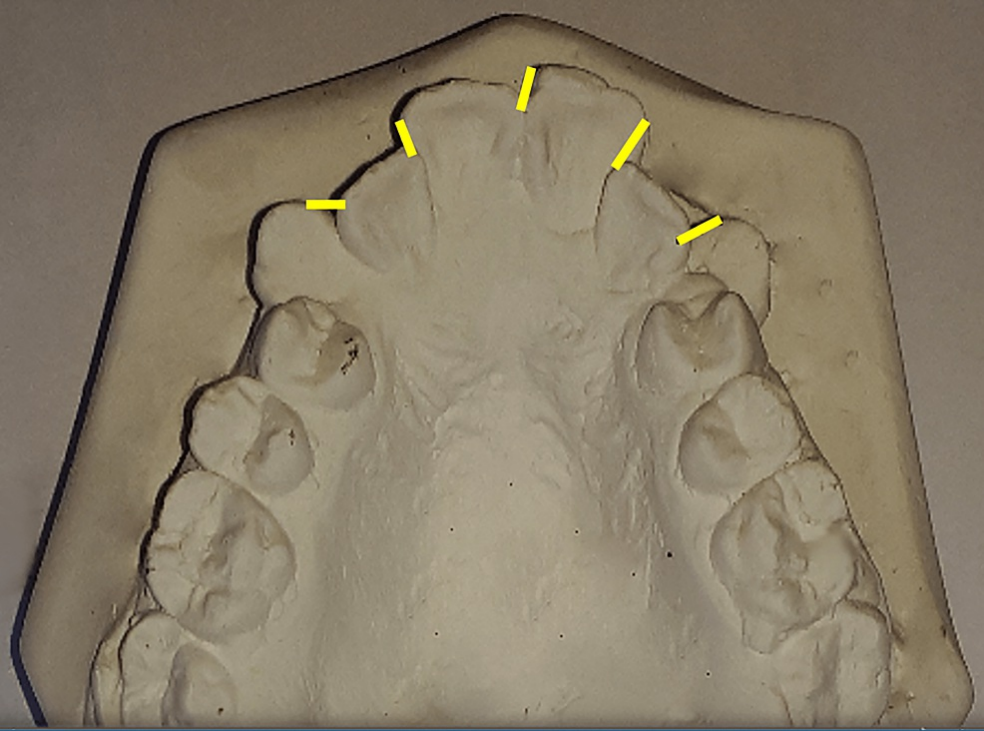
Measurement of Little's Irregularity Index (LII) on digital photographs Displacements between contact points (shown in yellow lines) are summed to obtain the LII score.

**Figure 3:**
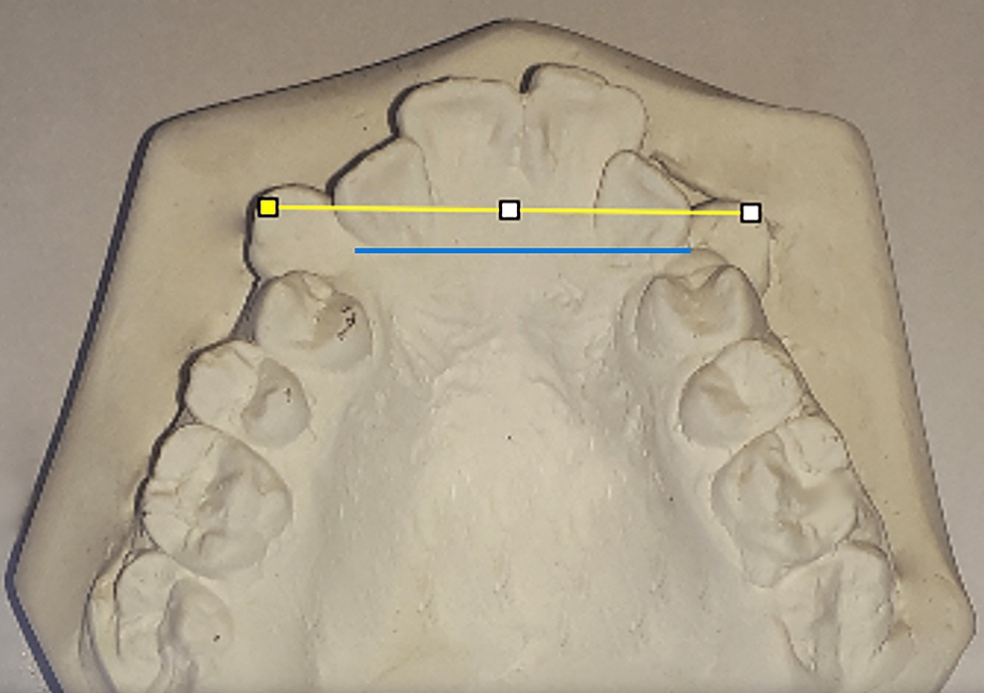
Measurement of intercanine width on digital photographs The yellow line represents the intercanine width at the cusps level, and the blue line represents the intercanine width at the cervical region from the lingual side.

**Figure 4:**
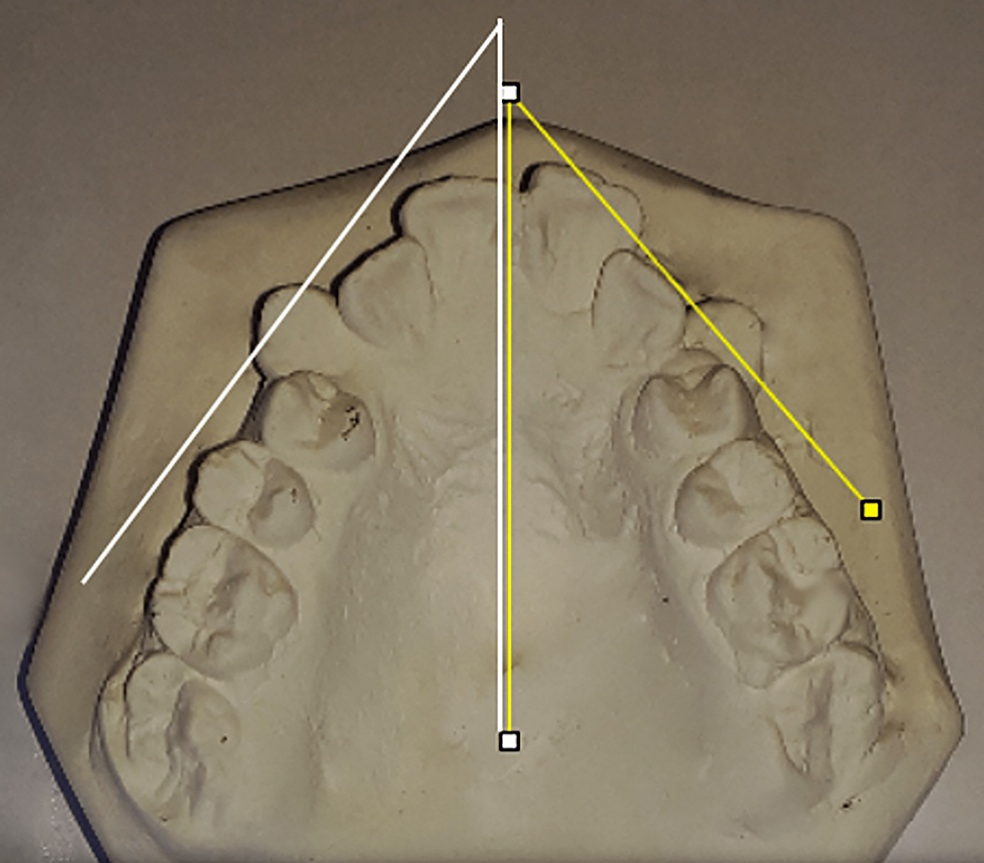
Measurement of the canine rotation angles on digital photographs The right canine rotation angle is formed by the white intersecting lines, whereas the left is formed by the yellow intersecting lines.

Dental casts were made at each assessment time, then digital photographs of these casts were taken. The reference points and levels were determined on the digital photographs; then, the measurements were calculated using Image J software. The Little's irregularity index values were calculated by measuring the horizontal distance between the maxillary six anterior teeth' mesial and distal contact points. The sums of the five measured distances represented the total value of irregularity (Figure 2). The intercanine width (lingual) was measured between the most lingual points of the upper canines while the intercanine width (cusp) was measured between the cusp tips of the upper canines (Figure 3). The canine rotation angle was formed between a line representing the midpalatal suture and another line going through the contact points of each upper canine (Figure 4).

Statistical analysis 

SPSS® Version 20 was employed to make the statistical analysis. The normal distribution of data was revealed using the Shapiro-Wilk test. The three groups' comparisons were achieved using the one-way ANOVA or Kruskal-Wallis test. The Bonferroni test as a post-hoc test or the Mann-Whitney test was used for pairwise comparisons. The results were considered significant at p ≤ 0.05 for all tests except for the Mann-Whitney test, where p ≤ 0.017 was the significance level, as it was modified according to the Bonferroni correction.

The error in the method

Twenty dental casts were randomly selected and remeasured by the principal researcher (H.M.I) after a one-month interval. The intra-examiner reliability (random error) was checked using interclass correlation coefficients, whereas paired-sample t-tests were used to detect systematic errors.

## Results

Baseline sample characteristics

Sixty-three participants (46 females, 17 males; mean age ± SD: 19.71 ± 2.74 years) participated in this study. Table 1 displays the baseline individuals' characteristics. There was no withdrawal from the study; therefore, the data analysis included all 63 participants.

**Table 1:** Basic sample characteristics (gender and age) n: number of patients, SD: standard deviation, Min.: minimum, Max.: maximum, Group 1: traditional brackets group, Group 2: self-ligating brackets group, Group 3: self-ligating brackets + flapless piezocision group; *employing chi-square test; ** age is given in years; †employing one-way ANOVA ANOVA: analysis of variance

Group	Gender	n (%)	P-value*	Mean age** (SD)	Min. age	Max. age	P-value†
Group 1 (n=21)	Male	5 (23.80 %)	0.674	20.72 (2.85)	18.30	27.10	0.954
	Female	16 (76.19 %)					
Group 2 (n=21)	Male	6 (28.57 %)		18.48 (2.96)	17.00	27.50	
	Female	15 (71.42 %)					
Group 3 (n=21)	Male	5 (23.80 %)		19.95 (2.39)	18.00	26.40	
	Female	16 (76.19 %)					
All sample (n=63)	Male	17 (26.98 %)		19.71 (2.74)	18.00	27.50	
	Female	46 (73.01 %)					

The error in the method 

The interclass correlation coefficients revealed strong intra-examiner reliability for the measures, ranging from 0.985 to 0.999. In addition, no significant differences between the first and second measurements were found using paired sample t-tests (P > 0.05), which indicated insignificant systematic errors.

Dentoalveolar changes 

At the T1, T2, and T3 assessment times, there were statistically significant differences between the three groups regarding LII (P < 0.001; Tables 2, 3). Conversely, no statistically significant differences were detected at the last assessment time between the three studied groups (P = 0.666; Table 4).

**Table 2:** Descriptive statistics of Little's irregularity index (LII) at five assessment times in the three groups and the results of significance test† † Employing one-way ANOVA test or Kruskal-Wallis test, SD: Standard Deviation, Min: minimum, Max: maximum, CI: confidence interval, T0: before the beginning of orthodontic treatment, T1: after 1 month, T2: after 2 months, T3: after 3 months, T4: at the end of the leveling and alignment phase, Group 1: traditional brackets group, Group 2: self-ligating brackets group, Group 3: self-ligating brackets + flapless piezocision group, NS: there was no statistically significant difference at P > 0.05, ***: there was a statistically significant difference at P < 0.001 ANOVA: analysis of variance

Time	Group	Mean	SD	Min	Max	95% CI for difference		P-value	Significance
						Lower bound	Upper bound		
T0	Group 1	12.28	1.16	10.00	14.40	11.73	12.82	0.466	NS
	Group 2	12.13	1.06	10.00	14.00	11.61	12.64		
	Group 3	12.58	1.19	10.60	14.70	12.00	13.16		
T1	Group 1	9.82	1.06	7.90	11.60	9.32	10.31	<0.001	***
	Group 2	8.06	0.60	6.90	8.80	7.77	8.35		
	Group 3	6.66	1.12	5.30	8.90	6.12	7.20		
T2	Group 1	7.00	0.92	5.50	8.90	6.56	7.43	<0.001	***
	Group 2	4.85	0.56	3.70	5.70	4.58	5.12		
	Group 3	2.68	0.69	1.00	3.90	2.34	3.02		
T3	Group 1	4.45	0.71	3.10	5.80	4.11	4.79	˂0.001	***
	Group 2	2.65	0.73	1.00	3.90	2.30	3.01		
	Group 3	0.42	0.76	0.00	2.10	0.05	0.79		
T4	Group 1	0.27	0.60	0.00	2.20	-0.00	0.55	0.666	NS
	Group 2	0.17	0.54	0.00	2.00	-0.08	0.44		
	Group 3	0.14	0.42	0.00	1.50	-0.06	0.34		

**Table 3:** Descriptive statistics of the intercanine widths and the canine rotation angle at two assessment times in the three groups and the results of significance test† † Employing one-way ANOVA test or Kruskal-Wallis test, SD: Standard Deviation, Min: minimum, Max: maximum, CI: confidence interval, T0: before the beginning of orthodontic treatment, T4: at the end of the leveling and alignment phase, Group 1: traditional brackets group, Group 2: self-ligating brackets group, Group 3: self-ligating brackets + flapless piezocision group, NS: there was no statistically significant difference at P > 0.05, **: there was a statistically significant difference at P < 0.01 ANOVA: analysis of variance

Time	Group	Mean	SD	Min	Max	95% CI for difference		P-value	Significance
						Lower bound	Upper bound		
Intercanine width at the cusps level									
T0	Group 1	34.23	0.91	33.00	36.40	33.80	34.66	0.477	NS
	Group 2	34.56	1.24	33.00	37.20	33.96	35.16		
	Group 3	34.81	1.34	33.00	36.90	34.16	35.46		
T4	Group 1	35.40	0.90	34.10	37.40	34.97	35.82	0.005	**
	Group 2	36.55	1.29	34.20	39.00	35.93	37.18		
	Group 3	36.73	1.69	34.20	39.00	35.92	37.55		
Intercanine width at the cervical level from the lingual side									
T0	Group 1	26.44	1.28	24.20	29.10	25.84	27.04	0.461	NS
	Group 2	26.95	1.38	24.80	29.80	26.28	27.62		
	Group 3	27.05	1.75	24.50	29.40	26.20	27.89		
T4	Group 1	27.27	1.31	25.10	29.70	26.65	27.88	0.329	NS
	Group 2	27.76	1.32	25.70	30.40	27.12	28.40		
	Group 3	27.93	1.63	25.20	30.20	27.14	28.71		
Canine rotation angle									
T0	Group 1	46.19	7.87	34.40	62.90	42.50	49.87	0.894	NS
	Group 2	37.02	11.40	13.70	52.90	31.52	42.52		
	Group 3	47.01	14.24	9.10	74.30	40.15	53.88		
T4	Group 1	41.61	3.31	34.70	47.50	40.06	43.16	0.592	NS
	Group 2	40.81	3.70	36.10	47.40	39.03	42.59		
	Group 3	41.61	3.61	36.30	49.30	39.87	43.35		

**Table 4:** Post-hoc tests for Little's Irregularity Index and the intercanine width (at the cusp level) in the pairwise comparisons† † Bonferroni was used to detect any significant difference between every two groups, LII: Little's Irregularity Index, CWC: intercanine width at the cusps level, T1: after 1 month, T2: after 2 months, T3: after 3 months, T4: at the end of the leveling and alignment phase, Group 1: traditional brackets group, Group 2: self-ligating brackets group, Group 3: self-ligating brackets + flapless piezocision group; NS: there was no statistically significant difference at P > 0.05, *: statistically significant at P < 0.05, **: statistically significant at P < 0.01, ***: statistically significant at P < 0.001

Variable	Time	Group comparison	Mean difference	95% CI for difference		P-value	Significance
				Lower bound	Upper bound		
LII	T1	Group 1 vs. Group 2	1.75	0.99	2.51	>0.001	***
		Group 1 vs. Group 3	3.15	2.39	3.91	>0.001	***
		Group 2 vs. Group 3	1.40	0.63	2.16	>0.001	***
	T2	Group 1 vs. Group 2	2.14	1.55	2.73	>0.001	***
		Group 1 vs. Group 3	4.31	3.72	4.90	>0.001	***
		Group 2 vs. Group 3	2.16	1.57	2.76	>0.001	***
	T3	Group 1 vs. Group 2	1.79	1.21	2.38	>0.001	***
		Group 1 vs. Group 3	4.02	3.44	4.61	>0.001	***
		Group 2 vs. Group 3	2.23	1.63	2.82	>0.001	***
CWC	T4	Group 1 vs. Group 2	1.15	0.10	2.21	0.027	*
		Group 1 vs. Group 3	1.33	0.28	2.39	0.008	**
		Group 2 vs. Group 3	0.17	-0.88	1.24	1.000	NS

Post-hoc tests at T1, T2, and T3 showed differences between all groups, and the mean greater values were in the self-ligating brackets with the flapless piezocision group. Regarding the intercanine width (lingual), no difference was detected between the three groups at the end of the leveling and aligning phase (T4, P = 0.329). On the other hand, the intercanine width (cusp) at T4 revealed greater mean values in the self-ligating brackets alone group and the self-ligating brackets with flapless piezocision group compared to the traditional brackets group, and these differences were statistically significant (P = 0.005). Concerning the canine rotation angle, there were no statistically significant differences between the three groups at the end of the leveling and alignment phase (P = 0.592).

## Discussion

This randomized controlled trial is the first one evaluating the dentoalveolar changes following flapless piezocision with self-ligating brackets compared to self-ligating brackets or traditional brackets alone in adult patients with anterior maxillary severe crowding. 

The mean values of LII revealed statistically significant differences between the three groups, where self-ligating brackets with the flapless piezocision group showed the greatest improvement rate in the index means values during the first three months. This result could be explained by the regional acceleratory phenomenon induced by the applied surgical intervention and the effect of the active self-ligating brackets used in the current study, which may result in better engagement between wires and brackets.

There was no difference between the three groups regarding the intercanine width (lingual) at the end of the leveling and aligning phase (P > 0.05). On the other hand, the intercanine width (cusp) showed greater mean values at the end of the leveling and alignment phase in both self-ligating brackets groups (i.e., alone or with flapless piezocision) in comparison with the traditional brackets group. This difference may be attributed to the disparity in the brackets system used, as the degrees of buccal inclination in the canines' self-ligating brackets were greater.

With regard to the changes in intercanine widths associated with flapless piezocision, no previous studies evaluated these outcomes. As for the comparison between the two types of brackets (traditional vs. self-ligating), the current results agreed with the Anand et al. study, which found a significant increase in the intercanine width in the self-ligating brackets group [24]. Conversely, the findings of this trial disagreed with those reported by Moyano et al.'s study, which did not find any significant differences between the two types of brackets [25,26]. Although Fleming et al. found that the intercanine width was lower in the self-ligating brackets group, these differences were not statistically significant [25]. This discrepancy in the results of the studies may be due to the different types of brackets used and, thus, the preprogrammed features of the bracket's slot.

There was no difference in the canine rotation angle between the three groups at the end of the leveling and alignment phase (P > 0.05). This could be explained by the nature of the canine's movements at this phase, which depended on the sequence of wires only without applying any additional orthodontic forces, such as using power chains; consequently, this might allow enough time for the canines to align properly without any rotation.

No previous studies have evaluated changes in canine rotation angle after orthodontic treatment associated with flapless piezocision. Furthermore, no trials have studied changes in canine rotation angle concerning using traditional brackets compared to self-ligating brackets in cases of dental crowding.

Throughout this study, no significant harm occurred, as patients did not report any important post-surgical side effects. The current findings may be generalized to individuals who receive comparable treatment interventions and meet similar inclusion criteria of this study.

Limitations

There are some limitations of the present randomized controlled trial. First, a detection bias is possible because patients and the principal researcher cannot be blinded. Second, this study did not follow up on the dentoalveolar changes for the entire period of orthodontic treatment. Third, the side effect of the surgical intervention was not assessed. Finally, dentoalveolar changes associated with different acceleration techniques are suggested in future studies.

## Conclusions

Using self-ligating brackets with flapless piezocision revealed more significant results concerning LII compared to other groups. Thus, combining these two acceleration methods could get more effective results in aligning severely crowded teeth. Self-ligating brackets, whether used alone or with flapless piezocision, resulted in a greater intercanine width at the level of cusps. The types of brackets (traditional or self-ligating) did not affect the canine rotation angle.
